# Dynamic retinal vessel analysis: flickering a light into the brain

**DOI:** 10.3389/fnagi.2024.1517368

**Published:** 2025-01-06

**Authors:** Anna Peterfi, Ana Clara da C. Pinaffi-Langley, Zsofia Szarvas, Mihaly Muranyi, Zalan Kaposzta, Cheryl Adams, Camila Bonin Pinto, Peter Mukli, Konstantin Kotliar, Andriy Yabluchanskiy

**Affiliations:** ^1^Oklahoma Center for Geroscience and Healthy Brain Aging, University of Oklahoma Health Sciences, Oklahoma City, OK, United States; ^2^Vascular Cognitive Impairment and Neurodegeneration Program, Department of Neurosurgery, University of Oklahoma Health Sciences, Oklahoma City, OK, United States; ^3^International Training Program in Geroscience, Doctoral School of Basic and Translational Medicine/Institute of Preventive Medicine and Public Health, Semmelweis University, Budapest, Hungary; ^4^Department of Nutritional Sciences, College of Allied Health, University of Oklahoma Health Sciences, Oklahoma City, OK, United States; ^5^Oklahoma Shared Clinical and Translational Resources, University of Oklahoma Health Sciences, Oklahoma City, OK, United States; ^6^Department of Medical Engineering and Technomathematics, Aachen University of Applied Sciences, Juelich, Germany; ^7^Department of Health Promotion Sciences, College of Public Health, University of Oklahoma Health Sciences, Oklahoma City, OK, United States; ^8^Peggy and Charles Stephenson Cancer Center, University of Oklahoma Health Sciences, Oklahoma City, OK, United States

**Keywords:** dementia, brain, dynamic vessel analysis, retinal vasoreactivity, aging

## Abstract

**Introduction:**

Growing aging populations pose new challenges to public health as the number of people living with dementia grows in tandem. To alleviate the burden of dementia, prodromal signs of cognitive impairment must be recognized and risk factors reduced. In this context, non-invasive techniques may be used to identify early changes and monitor disease progression. Dynamic retinal vessel analysis (DVA) provides an opportunity to measure retinal vasoreactivity in a way that may be comparable to cerebral vasoreactivity, thus providing a window to the brain.

**Methods:**

We conducted a literature search on PubMed and Scopus to identify studies utilizing DVA to describe retinal vasoreactivity in central nervous system diseases and compare it with brain function and structure. We included original papers with full text in English.

**Results:**

We identified 11 studies, of which most employed a cross-sectional design (91%). Studies on cerebrovascular diseases reported that retinal vasoreactivity decreased in patient populations compared with that of healthy controls. Studies on cognitive impairment and dementia yielded mixed results, at least in part due to high population heterogeneity. There is also evidence for the association between DVA and brain and cognition parameters such as cerebral blood flow velocity, cerebral microvascular diffusivity, and cognitive function score.

**Discussion:**

The reviewed papers on DVA and brain function, despite the mixed results, have demonstrated the relationship between retinal vasoreactivity and cerebrovascular function and cognition. Heterogeneity in study populations, procedures, and analyses make comparisons difficult. Studies with larger sample size, clear description of the population and methods, and standardized DVA analysis are needed to elucidate the eye–brain connection and to enhance the translational and clinical applications of DVA.

## Introduction

Aging populations around the world pose new challenges to public health and healthcare systems. In the latest report of the Global Burden of Disease (Institute for Health Metrics and Evaluation, 2021), an estimated 57 million people were living with dementia in 2021 worldwide, representing a 46% increase from 2010. By 2050, this number is expected to almost triple to 153 million people (GBD 2018 Dementia Forecasting Collaborators, 2022). Importantly, the most prevalent risk factors for dementia are cardiovascular risk factors: fasting hyperglycemia, obesity, smoking, and hypertension (Farnsworth von Cederwald et al., 2022; McGrath et al., 2022; Song et al., 2021; Chang Wong and Chang Chui, 2022). Thus, cardiovascular risk factor reduction and recognition of the prodromal signs of cognitive impairment are crucial for dementia prevention and to alleviate the burden of this disease.

The retina is part of the central nervous system and has the same embryological origin as the brain, and in some applications is considered as a “window to the brain” (Ptito et al., 2021). The eye has long been used to help diagnose and evaluate systemic diseases like diabetes (Lin et al., 2021), hypertension (Dziedziak et al., 2022), and thyroid gland-related diseases (Bojikian and Francis, 2019). This is one of the few organs where we can non-invasively see the small arteries and veins, as well as arterioles, venules and capillaries with our own eyes *in vivo*, even red blood cells on certain occasions. Several noninvasive and easily performed techniques provide information about the vascular and neuronal function and structure in the eye. Optical coherent tomography (OCT) produces images of the neuronal fibers and retinal layers. Optical coherent tomography angiography (OCTA), ophthalmoscopy and retinal angiography evaluate the health of the eye vasculature. Meanwhile electrophysiology tests (e.g., visual evoked potential, electroretinography) evaluate the neuronal functions of the retina and the visual pathway. A technique termed dynamic vessel analysis (DVA) produces videos of the retina and allows precise measurements of vessel diameter changes over time. Among other applications, this is used to evaluate functional vascular hyperemia responses upon photoreceptor stimulation with flickering light. DVA has been used in research in the context of several diseases, including eye (Selbach et al., 2014; Iacono et al., 2017), metabolic (Kotliar et al., 2011; Schiel et al., 2009), and vascular (Machalinska et al., 2018; Theuerle et al., 2021) pathologies, among others (Sharifizad et al., 2021; Rabiolo et al., 2016; Csipo et al., 2021).

Using DVA to evaluate brain health is of particular interest. Although more common techniques such as OCT and OCTA can be applied to study the connection between the eye, the brain, and cognitive function (Czako et al., 2020), DVA offers an important advantage in its capacity to measure functional hyperemia because this process can also be measured in the brain (Huneau et al., 2015; Csipo et al., 2019). Though, the exact role and importance of each contributor to functional hyperemia–endothelial cells, Müller glial cells, astrocytes, among others–is still under examination in the eye (Newman, 2013; Pournaras et al., 2008), DVA is a good technique to investigate the same response in a different tissue that shares a common origin with the brain. Moreover, pre-clinical and clinical studies show that impaired functional hyperemia in the brain is associated with worse cognitive performance (Mukli et al., 2024; Tarantini et al., 2017). However, whether functional hyperemia in the retinal vessels can be associated with brain and cognitive health remains underexplored. This narrative review aimed to identify and summarize available studies that employed DVA with its flicker stimulation to investigate brain function and cognitive performance in humans. We also aimed to describe areas for improving the translatability of future studies and the clinical relevance of the technique.

### DVA: brief technical background

**Figure 1:**
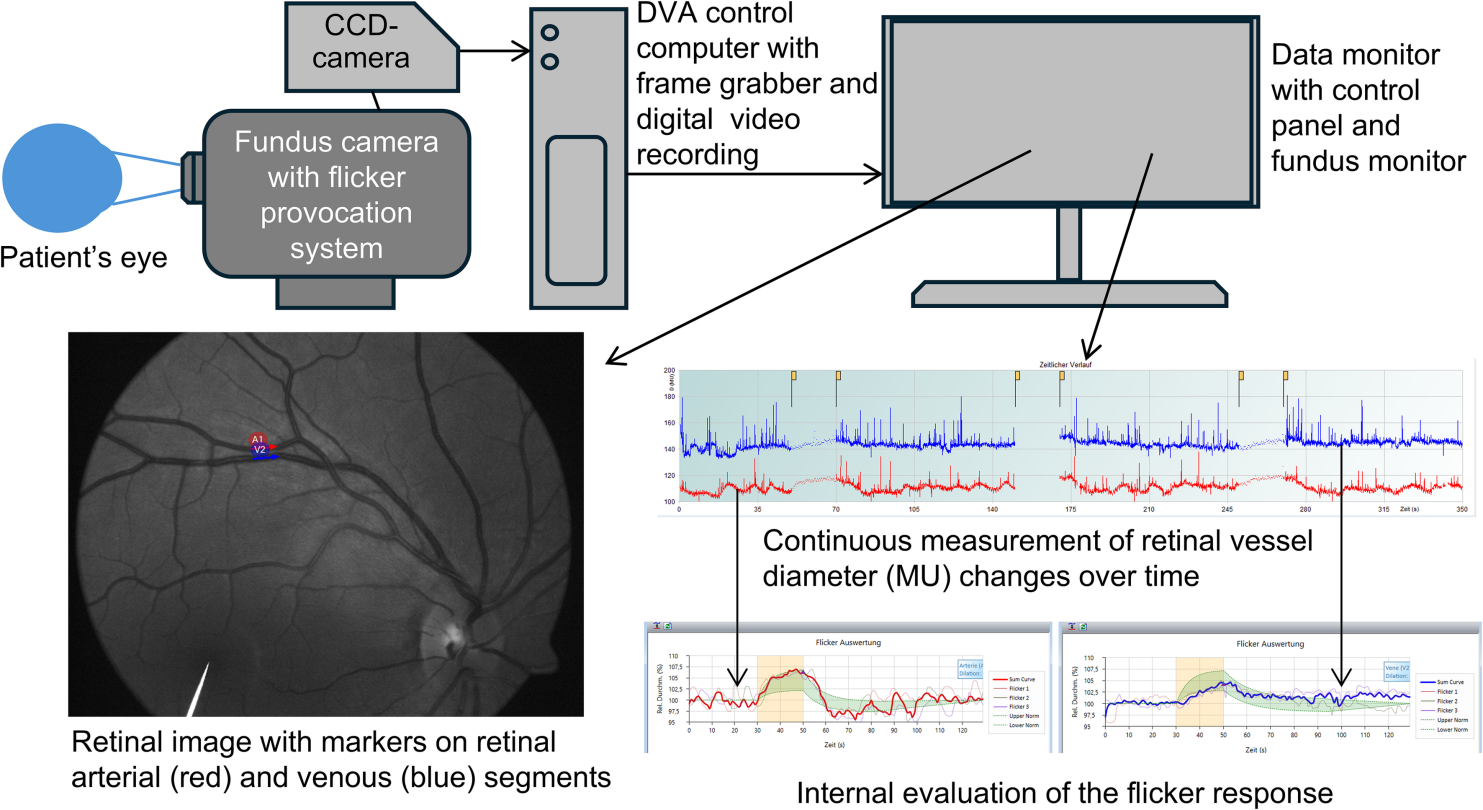
Simplified setup of the dynamic retinal vessel analysis system and representative findings. The image is reproduced with permission from the subject. CCD, charge-coupled device.

DVA involves illuminating the retina with a bright green light and collecting diameter values from selected vessels, small arteries and veins, branches of central retinal artery and vein, from high-definition image recordings (25 frames per second). The Dynamic Vessel Analyzer software (Imedos Health GmbH, Jena, Germany) is used to set the stimulation parameters and measurement duration, which typically follows the standard DVA protocol and lasts 350 s (Kotliar et al., 2011; Kotliar et al., 2017; Mandecka et al., 2007). During this time, a baseline period of steady bright green light is followed by alternating cycles of rectangular luminant flickering light (stimulus) at a frequency of 12.5 Hz and steady light (resting). Generally, the length of stimulation and rest, and the number of stimulation cycles can be set up in the software and vary greatly within published methodologies (see Discussion for further details). However, most studies use the mentioned standard DVA protocol: 50 s baseline, 20 s flickering period, 80 s recovery, 3 cycles, described in detail elsewhere (Kotliar et al., 2011; Kotliar et al., 2017; Albanna et al., 2016; Mandecka et al., 2007; Kneser et al., 2009). Using the recorded images, the software calculates baseline vessel diameters along with the maximal dilation and maximal constriction values, providing these results as a relative in percentage compared with the baseline value. Other available data include the raw individual diameter values, the smoothed data, and the average diameter per second. Some studies derive additionally more meaningful parameters of retinal vessel diameter response to flicker, e.g., times of maximal constriction and dilation, areas under the flicker response curves, etc. (Kotliar et al., 2011; Kotliar et al., 2017). We direct the reader to a consensus paper by Garhofer et al. (2010) for more details on how to use the DVA device. Figure 1 presents a simplified setup of modern DVA system and output.

## DVA and brain function

To identify published studies on DVA and brain function, we searched PubMed and Scopus for interventional and observational human studies using the terms “dynamic vessel analysis,” “brain,” “cognition,” “cognitive,” “flicker,” and “flickering” without publication year restrictions. The search yielded nine publications; of these, one was excluded because it was a conference abstract. Three papers were identified by screening the reference lists. Of the eleven studies, one was longitudinal, the others were cross-sectional, from which two utilized a population-based cohort (the Maastricht study). Table 1 summarizes the characteristics of the retrieved studies. Supplementary Table 1 details the comorbidities of participants reported in the retrieved studies.

**Table 1:** Characteristics of studies presented in this review investigating the relationship between retinal vasoreactivity and brain function.

Reference	Study design	Participant characteristics	Measurements	DVA parameters	Main findings
Bettermann et al. (2012)	Cross-sectional	Patients with chronic IWMD (*n* = 12; mean age 64 ± 11 years; 57% female), controls (*n* = 14; mean age 55 ± 8 years; 33% female)	DVA, TCD, MRI	50 s baseline, 20 s flickering period, 80 s recovery, 3 cycles	Patients had decreased retinal arterial (*p* = 0.004) end venous (*p* = 0.002) reactivity; attenuated retinal venous response was associated with significantly decreased middle cerebral artery reactivity (*r* = 0.453, *p* = 0.045)
Mroczkowska et al. (2014)	Cross-sectional	Patients with mild AD (*n* = 10; mean age 63 ± 8 years; 50% female), age-matched healthy controls (*n* = 28; mean age 58 ± 7 years; 39% female)	DVA	50 s baseline, 20 s flickering period, 80 s recovery, 3 cycles	Longer reaction time to maximal dilation of the artery during the first and third flickering period in patient group vs. controls (*p* = 0.038, *p* = 0.028 respectively), and shorter reaction time of the artery during the second flickering period in patient group vs. controls (*p* = 0.049). Negative correlation between the arterial reaction time of the first cycle and MMSE score in patients (*r* = −0.782, *p* = 0.013).
Albanna et al. (2016)	Cross-sectional	Patients with acute aSAH (*n* = 6; mean age 56 ± 16 years) and late aSAH (*n* = 8; mean age 47 ± 12 years), age-matched healthy controls (*n* = 33; mean age 51 ± 10 years). Sex distribution not reported	SVA, DVA	50 s baseline, 20 s flickering period, 80 s recovery, 3 cycles	Trend toward impaired vasodilatio*n* (*p* = 0.08) and vasoconstriction (*p* = 0.09) in acute aSAH group compared with healthy controls.
Bettermann et al. (2017)	Cross-sectional	Patients with prediabetes (*n* = 22; mean age 60 ± 10 years; 55% female), patients with T2DM (=37; mean age 58 ± 4 years; 51% female), and controls (*n* = 13; mean age 52 ± 9 years; 92% female)	SVA, DVA, TCD	50 s baseline, 20 s flickering period, 80 s recovery, 3 cycles	Impaired retinal arterial and venous dilation to flickering light in patients with T2DM compared with controls (retinal *p* = 0.003, venous *p* = 0.012). Impaired retinal venous dilation response in patients with prediabetes compared with controls (*p* = 0.004). Correlation between cerebral vascular (MCA) reaction and retinal venous response (*r* = 0.24, *p* = 0.046).
Kotliar et al. (2017)	Cross-sectional	Patients with ADD (*n* = 15; mean age 74 [67–80] years; 60% female); MCI (all *n* = 24; mean age 69 [64–73] years; 58% female): MCI-AD (*n* = 13), MC-nonAD (*n* = 11); and healthy controls (*n* = 16; mean age 69 [60–71] years; 62% female)	SVA, DVA	50 s baseline, 20 s flickering period, 80 s recovery, 3 cycles	Arterial dilation increased in ADD vs. HC (*p < 0.02) & MCI (*p = n.s.), increased in MCI-nonAD vs. HC (*p* = 0.03), decreased in MCI-AD vs. ADD (*p* = 0.041), delayed in ADD vs. HC (*p < 0.01) & MCI (*p < 0.02) [MCI-AD *p* = 0.007, MCI-nonAD *p* = 0.033]. Venous dilation increased in ADD vs. HC (*p < 0.01) & MCI (*p < 0.02), decreased in MCI-AD vs. ADD (*p* < 0.001) & MCI-nonAD (*p* = 0.041). Arterial constriction increased in MCI-AD vs. HC (*p* = 0.032) & ADD (*p* = 0.001).
Conzen et al. (2018)	Cross-sectional	Patients with acute aSAH (*n* = 24; mean age 54 ± 11 years; 71% female) and late aSAH (*n* = 11; mean age 52 ± 16 years; 82% female), age-matched healthy controls (*n* = 35; mean age 51 ± 9 years; 49% female)	SVA, DVA	50 s baseline, 20 s flickering period, 80 s recovery, 3 cycles	Acute aSAH group had decreased retinal arterial dilation (*p* < 0.001), AUC (*p* = 0.003) and longer time to maximum arterial (*p* = 0.023) and venous (*p* < 0.001) dilation compared with healthy controls. Late phase group had decreased retinal arterial dilation (*p* = 0.008), longer time to maximum arterial (*p* = 0.003) and venous (*p* < 0.01) dilation compared with healthy controls.
Querques et al. (2019)	Cross-sectional	Patients with AD (*n* = 12; mean age 73 ± 7 years; 67% female), MCI (*n* = 12; mean age 76 ± 7 years; 58% female), and controls (*n* = 32; mean age 72 ± 6 years; 47% female)	DVA, OCT, OCTA, cerebrospinal fluid sample	50 s baseline, 20 s flickering period, 80 s recovery, 3 cycles	Arterial dilation reduced in AD group vs. MCI (*p* = 0.045) and to controls (*p* = 0.002), amplitude of vessel reaction was reduced in AD (*p* < 0.0001) and MCI (*p* = 0.048) groups vs. controls, arterial dilation (*R* = 0.441, *p* = 0.04) and reaction amplitude (*R* = 0.58, *p* = 0.005) were correlated with amyloid *β* level.
Rensma et al. (2020)	Cross-sectional	Population-based cohort (*n* = 3,011; mean age 60 ± 8 years; 49% female)	Several microvascular measurements, DVA, cognitive performance (memory, information processing speed, executive function)	50 s baseline, 40 s flickering period, 60 s recovery, 1 cycle	Microvascular damage score is associated with worse cognitive score (*p* < 0.05), DVA alone did not show connection with cognitive performance.
Szegedi et al. (2020)	Cross-sectional	Patients with AD (*n* = 23) and MCI (*n* = 24) (mean age 73 ± 9 years; 40% female), and controls (n = 43; mean age 71 ± 7 years; 53% female)	OCT: RNFL thickness, central retinal thickness, retinal blood flow; retinal oxygen saturation, SVA, DVA	60 s baseline, 60 s flickering period, 1 cycle	Flicker induced vasodilation showed no difference.
Albanna et al. (2021)	Longitudinal (follow-up at 6 weeks; phone call at 12^th^ month)	Patients with aSAH (*n* = 70; mean age 51 [48–59] years; 73% female), age-matched healthy controls (*n* = 42; mean age 50 [43–59] years; 48% female)	SVA, DVA	50 s baseline, 20 s flickering period, 80 s recovery, 3 cycles	Retinal arterial dilation and AUC decreased in all aSAH phases except follow-up vs. healthy controls (*p* < 0.05). Venous AUC showed significant increase from acute phase to follow-up (*p* = 0.0205). aSAH patients with DCI had faster arterial dilation in acute phase vs. non-DCI patients (*p* = 0.022), then had slower arterial dilation and took longer time reaching maximum in late phase (*p* = 0.017).
van Dinther et al. (2022)	Cross-sectional	Subgroup of population-based cohort (*n* = 70; mean age 60 ± 8 years; 41% female)	T1-weighted and FLAIR images, and IVIM MRI, SVA, DVA	50 s baseline, 40 s flickering period, 60 s recovery, 1 cycle	Retinal arterial dilation showed positive association with IVIM-derived microcirculatory diffusivity in NAWM (*r* = 0.283, *p* = 0.02) and CGM (*r* = 0.295, *p* = 0.01).
Csipo et al. (2024)	Cross-sectional	Adults (*n* = 49; mean age 51 ± 20 years; 53% female)	FMD, LSCI, PWA, CANTAB, SVA, DVA	100 s baseline, 20 s flickering, 80 s recovery period, 3 cycles	After age adjustment, retinal arterial dilation correlated with reaction time (*r* = −0.433, *p* = 0.006). Retinal venous dilation correlated with reaction time (*r* = −0.352, *p* = 0.028) and with accuracy in rapid visual information processing task (*r* = 0.343, *p* = 0.033). Peripheral plus retinal vascular health score component 2 correlated with cognitive score component 3 (*r* = −0.453, *p* = 0.034) and 4 (*r* = −0.48, *p* = 0.024). Peripheral plus retinal vascular health score component 3 correlated with cognitive score component 2 (*r* = −0.049, *p* = 0.049) and with cognitive score component 4 (*r* = 0.424, *p* = 0.049).

AD, Alzheimer’s disease; ADD, Alzheimer’s disease dementia; aSAH, aneurysmal subarachnoid hemorrhage; CANTAB, Cambridge Neuropsychological Test Automated Battery; CGM, Cortical gray matter; DCI, Delayed cerebral ischemia; DVA, Dynamic vessel analysis; FLAIR, Fluid-attenuated inversion recovery; FMD, flow-mediated dilation; HC, Healthy controls; IVIM, Intravoxel incoherent motion; IWMD, Ischemic white matter disease; LSCI, laser speckle contrast imaging; MCA, middle cerebral artery; MCI, Mild cognitive impairment; MCI-AD, Mild cognitive impairment with AD biomarkers; MCI-nonAD, Mild cognitive impairment without AD biomarkers; MMSE, Mini-mental state examination; MRI, Magnetic resonance imaging; NAWM, Normal appearing white matter; OCT(A), Optical coherence tomography (angiography); PWA, pulse wave analysis; RNFL, Retinal nerve fiber layer; SVA, Static vessel analysis; TCD, Transcranial doppler; T2DM, Type 2 diabetes mellitus. *p corrected for multiple comparison.

## DVA and cerebrovascular function

Magnetic resonance imaging (MRI) offers a good opportunity for evaluating cerebrovascular function. However, this imaging modality is limited to the macrovasculature and the consequences of vascular pathologies, while microvascular changes remain imperceptible (Stringer et al., 2021). In this context, the intravoxel incoherent motion (IVIM) MRI was proposed to assess the brain microvasculature, allowing the detection of blood flow and fast directional changes in small cerebral vessels (Paschoal et al., 2018). Importantly, lower microvascular perfusion in the normal appearing white matter and grey matter measured by IVIM MRI were associated with lower cognitive performance in patients with small vessel disease (Zhang et al., 2017). A subgroup from the population-based cohort of the Maastricht study underwent IVIM MRI and the scan findings were compared with retinal vasoreactivity results (van Dinther et al., 2022). The authors reported that the retinal arterial dilation to flicker stimulus was associated with the pseudo-diffusion in the normal-appearing white matter and the cortical gray matter (adjusted to age, sex, and cardiovascular risk factors). The microcirculatory diffusivity is depending on the microvascular blood flow velocity and architecture of the microvascular bed in the normal-appearing white and cortical matter (van Dinther et al., 2022). With these results, the authors demonstrated that retinal vessel function and cerebral microvasculature properties are related and, with additional longitudinal investigations on a larger population, retinal vasoreactivity is a promising assessment to evaluate cerebral microvascular function (van Dinther et al., 2022).

## DVA and cerebrovascular diseases

### Chronic ischemic white matter disease

Chronic ischemic white matter disease (IWMD) is a steppingstone in the development of stroke and dementia (Chang Wong and Chang Chui, 2022). Using the eye for detecting early, subclinical signs of IWMD could help prevent or delay the progression of small vessel disease to dementia. A small pilot study found that people with chronic IWMD had decreased retinal arterial and venous reactivity to flickering light stimulus compared with healthy controls (Bettermann et al., 2012). The attenuated retinal venous response was significantly associated with decreased middle cerebral artery reactivity, which was defined as change in flow velocity in response to hyperventilation (breath-hold maneuver), measured by transcranial Doppler sonography. This association persisted after adjustment for age and vascular risk factors. However, the retinal arterial response was not correlated with the middle cerebral artery reactivity. Decreased arterio-venous ratio in the eye around the papilla was also significantly associated with decreased cerebral vasoreactivity in the IWMD group. The smaller arterio-venous ratio was due to increased venous diameters (Bettermann et al., 2012), which is associated as a significant risk factor for stroke (Girach et al., 2024). Importantly, some of the participants were administered phenylephrine for pupil dilation, which is an alfa-receptor agonist and may affect the arteries in the eye and thus influence their diameter and hence the results of DVA measurements (Lanzl et al., 2002; Bettermann et al., 2012).

### Acute aneurysmal subarachnoid hemorrhage

Acute aneurysmal subarachnoid hemorrhage (aSAH) can be followed by delayed cerebral ischemia (DCI) in 20–30% of cases, which worsens the prognosis of these patients; therefore, it is crucial that the appropriate therapy is administered in a timely manner (Ikram et al., 2021). A research group conducted cross-sectional (Albanna et al., 2016; Conzen et al., 2018) and prospective observational (Albanna et al., 2021) studies with aSAH patients and age-matched controls. They investigated whether DVA is a feasible assessment of the diameter and function of retinal vessels in this population and whether the technique could be used as a tool for predicting and monitoring the vascular outcomes of patients with aSAH. First, they recruited separate patients in the acute (within 2–14 days (Albanna et al., 2016) and 5–14 days (Conzen et al., 2018) of aSAH) and late [5 ± 2 months (Albanna et al., 2016) and 3 months (Conzen et al., 2018) after aSAH] phases and compared them with healthy age-matched controls in their cross-sectional studies. Interestingly, these two studies found different results in some parameters. The first pilot cross-sectional study (Albanna et al., 2016) found significantly dilated retinal arteries in aSAH patients compared with controls, which persisted when comparing the controls with patients in the late phase, albeit with a smaller but still significant difference. They also found a trend toward impaired arterial vasodilation and-constriction in the acute patient group compared with healthy controls, although this did not reach statistical significance. This impairment was attenuated in patients in the late phase (Albanna et al., 2016). The later cross-sectional study (Conzen et al., 2018) showed that arterial and venous diameters were decreased in the acute phase group compared with those of healthy controls, a difference that lessened to a trend in the late phase group, showing no more significance. The acute phase group had decreased retinal arterial dilation and area under the flicker response curve (AUC) as well as increased time to reach the maximal arterial and venous dilation compared with the healthy control group. Similarly, the late phase group also showed decreased arterial dilation and longer time to reach the maximal arterial and venous dilation than their healthy counterparts, although AUC values were not significantly different between the groups (control vs. late phase SAH: median arterial AUC 85.7 [interquartile range (IQR) 56.8–102.9] vs. 59.9 [IQR 38.5–106.9] %.s; median venous AUC 101 [IQR 81.7–128.1] vs. 91.1 [IQR 43.6–101.8] %.s) (Conzen et al., 2018).

Finally, the research group reported on the findings of a longitudinal study (Albanna et al., 2021) in which 70 patients with aSAH were followed up for more than 6 weeks after primary lesion occurrence. Patients in all phases after SAH had narrower retinal arteries and veins compared with healthy controls. During the acute phase, patients had decreased retinal arterial dilation and AUC compared with their healthy counterparts (control vs. aSAH: median arterial AUC 51.4 [IQR 32.5–69.7] vs. 21.5 [IQR 9.4–35.8] %.s) while the vessel dilation time course remained unchanged. At follow-up, these initial differences were no longer observed. From parameters related to venous reactivity, only the AUC showed a difference between phases, increasing at follow-up (52.4 [IQR 34.2–61.7] %.s) compared with the acute phase (41.3 [IQR 16.4–46.8] %.s). This study also reported differences between patients who did and did not develop DCI after SAH. During the acute phase, patients who later developed DCI had faster arterial dilation compared with those who did not develop DCI. This difference changed its direction in the late phase, when patients with DCI had an increased time to reach the maximal vessel dilation after stimulus (Albanna et al., 2021).

Patients with aSAH often receive nimodipine treatment, which is an L-type Ca^2+^ channel antagonist and may affect vessel reactivity. However, in the longitudinal study (Albanna et al., 2021), the authors found no evidence that the drug influenced the vessel reactivity parameters. Taken together, these studies demonstrated that DVA is feasible in patients with aSAH, and may work as a simple, non-invasive monitoring technique across different phases of the disease. However, further investigations are needed to place this measurement in the clinical setting.

### Stroke

Stroke risk is increased for people with diabetes as hyperglycemia injuries blood vessels, causing changes in microvascular behavior that are evident in both retinal and cerebrovascular vessels (Kim et al., 2008; Mandecka et al., 2007). Therefore, a study investigated whether the responsiveness of the retinal and cerebrovascular vessels is correlated in participants with prediabetes and type 2 diabetes (Bettermann et al., 2017). The study used DVA to assess the retinal vascular response to flickering light stimuli, and transcranial Doppler sonography to measure middle cerebral artery reactivity at rest and after a hyperventilation/breath-hold maneuver. They found that patients with prediabetes had attenuated retinal vascular reaction to flickering light stimulation compared with controls, whereas patients with type 2 diabetes had a decrease in both arterial and venous response after stimulation compared with controls. The authors also reported that cerebral vessel reactivity was directly correlated with retinal venous vasodilation in these patients. These results persisted after adjusting for age, hypertension, and other comorbidities (Bettermann et al., 2017). However, the authors reported that around half of the participants with type 2 diabetes needed extra phenylephrine eye drops for pupil dilation, which may affect the tone of the retinal arteries and influence the results of DVA. Further, it is unclear whether DVA results were adjusted for systemic blood pressure even though they reported continuously monitoring blood pressure during the measurements (Bettermann et al., 2017).

## DVA and cognitive function and dementia

### Alzheimer’s disease

Alzheimer’s disease (AD) is considered a neurodegenerative disease, but emerging evidence shows that it is a multifactorial disease with vascular contributions (Mehta and Mehta, 2023; Dumitrascu et al., 2024), with preclinical evidence indicating that endothelial dysfunction precedes the accumulation of amyloid beta plaques (Waigi et al., 2024). Although structural changes of AD and related dementias are detectable in the retina, such as the thinning of the peripapillary retinal nerve fiber layer and the macula, decreasing complexity of the vasculature, increased tortuosity (Czako et al., 2020), and amyloid plaque accumulations around the periarteriolar region (Dumitrascu et al., 2024), whether functional impairments in vasoreactivity can also be detected in the retina in early stages of cognitive impairment and dementia remains unclear. Kotliar et al. (2017) used DVA to investigate the differences in retinal reactivity in patients with AD dementia (ADD), mild cognitive impairment (MCI), and cognitively intact controls. The MCI group was divided to two additional groups based on the presence (MCI-AD) or absence (MCI-nonAD) of AD biomarkers for certain analysis. They found that both arterial and venous dilation was increased in the ADD group compared with the MCI and control groups. The reaction amplitude of the arteries both in the ADD and MCI groups were enhanced compared with controls. Additionally, they observed that the course of events during the DVA procedure was different in patients with ADD compared with patients with MCI and controls, with delayed arterial dilation and no rapid constriction following stimulation. In contrast, patients with MCI-AD exhibited stronger constrictions after the flickering light stimulation compared with controls and the ADD group, however patients with MCI-AD had decreased both arterial and venous dilation compared with patients with ADD, and decreased venous dilation compared with MCI-nonAD group. Lastly, the MCI-nonAD group showed increased arterial dilation compared with controls (Kotliar et al., 2017). Although these results were surprising, the authors suggest that the enhanced arterial and venous dilations and delayed vessel responses in MCI and ADD are related to neuronal components that are known to be impaired in AD. Moreover, it has been shown that the blood-oxygen-level-dependent (BOLD) response in the brain of patients with AD is also delayed (Rombouts et al., 2005).

Interestingly, Querques et al. (2019) found contrasting results when using the same DVA measurement protocol to assess arterial reactivity in patients with AD, MCI, and healthy controls. They reported that arterial dilation was decreased during flickering light stimulus in the AD group, and that the reaction amplitude was smaller in the AD and MCI groups compared with that observed in the control group. They also assessed amyloid-beta levels in the cerebrospinal fluid of participants and reported a direct correlation between amyloid-beta levels and arterial dilation and reaction amplitude (Querques et al., 2019). Importantly, some concerns regarding the characterization of the cohorts in this study warrant caution when interpreting their findings: the AD and MCI groups were not well characterized in terms of disease stage and underlying disorder, and the prevalence of hypertension was specified in the AD and MCI groups, but not in the control group. Hypertension influences retinal arterial reactions, representing an important confounder (Machalinska et al., 2018; Peregud-Pogorzelska et al., 2020).

A third study led by Szegedi et al. (2020) also investigated retinal vessel diameter reactivity to flickering light in patients with AD, MCI, and healthy controls. The authors reported no significant differences in retinal reactivity between patient and control groups. However, it is important to note that they did not define the maximal dilation calculation in their report. Lastly, a brief report authored by Mroczkowska et al. (2014) investigated the association between mild AD and retinal vasoreactivity. Their results showed that the arterial reaction of patients with mild AD was slower during the first and third (last) flickering period compared with that of healthy controls. In contrast, the arterial reaction was quicker during the second flickering period in the mild AD group compared with the control group. Furthermore, the arterial reaction time during the first flickering period was negatively correlated with the Mini Mental State score in patients with mild AD, but no correlation was observed with the following two flickering periods. The authors did not discuss possible explanations for these different associations between cognitive performance and flickering periods. These disparate results reflect the heterogeneity in DVA studies. For instance, while three of the four studies used the same DVA protocol (Kotliar et al., 2017; Querques et al., 2019; Mroczkowska et al., 2014), the third study administered a different protocol with only one 60-s baseline cycle and then one 60-s flickering light stimulation period (Szegedi et al., 2020). Further complicating direct comparisons between the studies, that each of them analyzed the DVA data with a different method. Finally, only one of the studies attempted to correlate retinal vasoreactivity with cognitive performance. Future studies on DVA and ADD should characterize their patients in terms of disease stage and comorbidities as these may affect flicker responses and contribute to the heterogeneity in study findings.

### DVA and cognitive performance

In a large population-based cohort study (the Maastricht study), researchers devised a microvascular damage score based on several microvascular measurements, including DVA. This score was compared with cognitive performance assessed with a neuropsychological battery in over 3,000 adults (Rensma et al., 2020). The authors found that the microvascular damage score showed a significant inverse association with cognitive performance (i.e., a higher microvascular damage score was associated with a lower cognitive performance score). However, when they analyzed retinal vasodilation response alone, they found no correlation with cognitive performance (Rensma et al., 2020).

Recently, a pilot cross-sectional study investigated the relationship of peripheral and retinal vascular health with cognitive performance (Csipo et al., 2024). This study utilized macro-and microvascular endothelial function measurements, static retinal vessel analysis, and DVA to assess vascular health, and a cognitive battery to assess cognitive performance in domains such as memory, attention, processing speed, and reaction time. When comparing DVA parameters with cognitive performance, age-adjusted analyses showed that retinal arterial and venous dilation were significantly associated with reaction time, and retinal venous dilation was significant correlated with accuracy in the rapid visual processing task. Next, the authors compared cognitive performance parameters with vascular health composite scores with and without retinal vessel measurements as loading variables in the principal component analysis. Interestingly, the authors reported more significant associations with cognitive performance parameters when retinal vascular analyses were included in the composite scores, indicating the importance of including retinal vessel analyses when investigating vascular health and cognitive performance.

## Discussion

In this review, we presented and discussed several studies that investigated the connection between retinal vasoreactivity using DVA and brain function. Although the significance of connection remains unclear, most studies reported changes in retinal vasoreactivity in disease states, thereby meriting larger studies designed to elucidate the many different factors that influence retinal vasoreactivity in health and disease. Several questions remain that limit the clinical utility of DVA: (i) What is the normal range of retinal vessel vasodilation as a response to flickering light stimulation? (ii) How does systemic blood pressure affect retinal responsiveness to stimulation? (iii) What is the best approach to analyze DVA data? (iv) Are there other methodological approaches to investigate brain function using DVA technology that can give us a clue to brain health? Regarding the first question, Streese et al. (2021) conducted a study with 277 participants to assess the normal dilation and constriction responses across different age groups in healthy participants aged between 20 and 82 years old. They reported that the maximal arterial dilation induced by flickering light stimulation did not change significantly with age, whereas the venous dilation response to stimulation decreased with increasing age. They also reported that arterial and venous vessel diameters decreased with aging. However, the number of participants in the study is too small for generating normative data and larger, population-based studies are needed to answer this question. Other studies investigating the association between age and DVA parameters found that the retinal arterial constriction was significantly decreased in older compared with younger individuals (Kneser et al., 2009; Seshadri et al., 2016). A study performed by our group reported decreased maximal retinal arterial dilation to flickering light stimulation in older compared with young participants (Lipecz et al., 2019).

Regarding the effect of blood pressure on retinal vasoreactivity, the above mentioned Streese et al. (2021) explained the unchanged arterial response with age with the increasing blood pressure in the older population. The higher blood pressure was also associated with the decreasing vessel diameters. Additionally, blood pressure and the central retinal arterial equivalent (summarized diameters of the arteries around the papilla) were positively and negatively associated, respectively, with flickering light-induced retinal arterial vasodilation (Streese et al., 2021). Another study by the same group showed that exercise-induced increase in blood pressure during DVA (in between baseline and flickering light period) increased the maximal retinal arterial dilation, and that healthy athletic individuals have a smaller baseline arterial diameter (Streese et al., 2021). However, it is important to distinguish chronic high blood pressure (uncontrolled hypertension) from acute higher blood pressure (e.g., due to stress and nervousness from the measurement, or due to exercise), as the former causes permanent narrowing and arteriosclerosis in the wall of the retinal arteries/arterioles over time (Cheung et al., 2022). Other studies that investigated the effect of blood pressure during or before DVA on retinal vessel diameters and vasoreactivity to flickering light in healthy individuals found no significant association (Nagel et al., 2006; Garhofer et al., 2003; Sorensen et al., 2017). Finally, while some studies (Bettermann et al., 2017; Bettermann et al., 2012) presented in this review collected blood pressure data and used it to normalize DVA results, this is not standard practice as continuous blood pressure monitoring requires an additional device connected to the DVA system. However, because of the above-mentioned reasons, recording the blood pressure at least at the beginning and at the end of the DVA measurement is advised by the manufacturer (Vilser, 2019).

Regarding analytical approaches, there are several differences between reports, contributing to the variety in results within this topic and precluding direct comparisons between studies on the same topic. Analyses differ in baseline length, maximal vascular dilation and constriction method of calculation, use of absolute or mean diameter values, and parameters calculated from raw diameter values. Moreover, descriptions of analytical procedures, data preprocessing, and calculations are not always reported in sufficient detail. Some research groups have attempted to provide solutions for standardizing the protocol and analysis of DVA measurements (Streese et al., 2021; Kotliar et al., 2011), but no widely accepted standard currently exists. Kotliar et al. (2011) have reported perhaps the most thorough DVA protocol in their methodology paper, which was used by some of the reviewed papers (Albanna et al., 2016; Kotliar et al., 2017). One reviewed study used a different approach, which is called sequential and diameter response analysis (Mroczkowska et al., 2014). This method considers the fluctuation of the baseline diameter and separates the analysis of the 3 cycles, resulting in additional variables, such as baseline-corrected flicker response and baseline diameter fluctuation next to the commonly used maximal dilation, constriction and other variables explaining the time course (Heitmar et al., 2010). Adjustment of the dilation with the baseline diameter was proposed by Nagel and colleagues, and although their results did not offer significant evidence for the effect of baseline diameter on maximal dilation (Nagel et al., 2004), other studies found a connection between baseline diameters and stimulation-induced maximal vessel dilation in small (Streese et al., 2021) and large vessels (Rembold et al., 2003). Of note, the inventors of DVA technology advise utilizing signal averaging when performing functional analyses of endothelial response to flicker stimulation (Vilser, 2019). This smoothing processing makes the DVA signal more stable and, where feasible, corrects inaccuracies and measurement errors.

The papers described in this review did not utilize the baseline-corrected flicker response analysis method except for Mroczkowska et al. (2014). Along with the variations in analytical approaches, we also observed considerable variations in the stimulus protocol. Table 1 includes the DVA protocol parameters used by each study. The flickering duration, recovery period, and number of cycles varied among studies. These differences may influence the physiological response induced with the flickering light and may diminish the meaning of comparisons between studies, ultimately affecting the clinical utility potential of DVA.

Finally, alternative methodological approaches may offer additional advantages compared with the most used flickering light assessment. Notably, flickering light-induced vasoreactivity must account for the baseline vessel diameter, which varies depending on systemic blood pressure (Dziedziak et al., 2022; Tapp et al., 2019) and physical activity (Streese et al., 2019), among other factors. Furthermore, the specific effect of the baseline diameter on the features of the dilation and the underlying mechanisms are still not fully understood. Thus, for flickering light-induced reactivity to truly reflect brain function, it must be adjusted accordingly. To gain further insight into the physiological and pathophysiological processes at play, it is essential to assess the retinal vessel behavior in greater depth. To overcome the limitations of the current approach and achieve more accurate results, it would be beneficial to consider additional aspects of retinal vessel dynamics and include complementary assessments such as oscillations and vasomotions. An animal study using an Alzheimer’s mice model reported that increased arterial vasomotions in mouse brain were associated with paravascular clearance (van Veluw et al., 2020), which is comparable with human studies utilizing DVA in patients diagnosed with AD reporting enhanced retinal arterial vasomotion compared with healthy individuals (Kotliar et al., 2022) and positively correlating arterial pulsation amplitude with neocortical amyloid beta scores (Golzan et al., 2017). Investigating pulsations and vasomotions of retinal vessels requires the assessment of the corresponding magnitudes, periodicity of the oscillations, their spectral characteristics, and wave forms, which can indirectly provide valuable information about vascular stiffness and the contractile state of the selected vessels (Kotliar et al., 2022; Vilser et al., 2002).

Another alternative methodological approach is combining other retinal imaging techniques with DVA to improve the visualization of vascular pathologies in the retina. Although the possibility of generating compact scores using static analyses such as OCT, OCTA, and static vessel analysis could simplify the interpretation of comprehensive vascular assessments, it is unclear how DVA parameters correlate with those obtained with other retinal imaging techniques. Among the studies summarized in this review, only one compared DVA with another retinal imaging method (OCT). Szegedi et al. (2020) reported no significant association between vessel reactivity and retinal nerve fiber layer thickness.

In conclusion, DVA provides an accessible method for non-invasive functional measurement of the retinal vasoreactivity. As part of the central nervous system, the retina has potential to become a surrogate tissue to assess cerebrovascular function and pathology. However, there is still a long road ahead to elucidate details of the relationship between retinal and cerebral functional vascular reactivity and how it relates to brain health. Methodological consensus as well as larger studies are needed to further promote the clinical utility of DVA.
