# Supplementary material for: Dynamic retinal vessel analysis: flickering a light into the brain
Source: Front Aging Neurosci. 2025 Jan 6;16:1517368. doi: 10.3389/fnagi.2024.1517368 (PMC11743452; doi:10.3389/fnagi.2024.1517368)
Supplement: Supplementary file 1 [file Table_1.docx]

Supplementary Table 1. Reported comorbidities of participants in the reviewed studies

| **Reference** | **Comorbidities (%)** |
| --- | --- |
| Bettermann et al. (2012) | T2DM: IWMD 50%, control 0%  Hypertension: IWMD 67%, control 0%  Dyslipidemia: IWMD 100%, control 57% |
| Mroczkowska et al. (2014) | NR |
| Albanna et al. (2016) | NR |
| Bettermann et al. (2017) | Hypertension medication: PWT2DM 93%, PWPD and control NR  Statin medication: PWT2DM 60%, PWPD and control NR |
| Kotliar et al. (2017) | T2DM: AD 0%, MCI 8%, HC 0%  Arterial Hypertension: AD 69%, MCI 42%, control 40% |
| Conzen et al. (2018) | NR |
| Querques et al. (2019) | Arterial hypertension: AD 64%, MCI 46%, control NR  CVD: AD 46%, MCI 64% control NR |
| Rensma et al. (2020) | T2DM: 27%, hypertension 55%, dyslipidemia 35%, prior CVD 16%, depression 4% |
| Szegedi et al. (2020) | Antihypertensive medication: AD and MCI 49%, control 44% |
| Albanna et al. (2021) | Hypertension: aSAH patients 50%, controls NR |
| van Dither et al. (2022) | Diabetes 42%, hypertension 63%, hypercholesterolemia 90%, history of CVD 13% |
| Csipo T et al. (2024) | Hypertension: 27%, diabetes 4%, thyroid hormone replacement 12%, estrogen hormone replacement 6% |

T2DM: type 2 diabetes mellitus, IWMD: ischemic white matter disease, NR: not reported, PWT2DM: patients with type 2 diabetes mellitus, PWPD: patients with prediabetes, AD: Alzheimer’s disease, MCI: mild cognitive impairment, CVD: cardiovascular disease, aSAH: acute subarachnoid hemorrhage

References

Albanna, W., Conzen, C., Weiss, M., Clusmann, H., Fuest, M., Mueller, M., et al. (2016). Retinal Vessel Analysis (RVA) in the Context of Subarachnoid Hemorrhage - A Proof of Concept Study. PLoS One 11, e0158781. doi: 10.1371/journal.pone.0158781.

Albanna, W., Conzen, C., Weiss, M., Seyfried, K., Kotliar, K., Schmidt, T. P., et al. (2021). Non-invasive Assessment of Neurovascular Coupling After Aneurysmal Subarachnoid Hemorrhage: A Prospective Observational Trial Using Retinal Vessel Analysis. Front. Neurol. 12, 690183. doi: 10.3389/fneur.2021.690183.

Bettermann, K., Slocomb, J. E., Shivkumar, V., and Lott, M. E. (2012). Retinal vasoreactivity as a marker for chronic ischemic white matter disease? J. Neurol. Sci. 322, 206-210. doi: 10.1016/j.jns.2012.05.041.

Bettermann, K., Slocomb, J., Shivkumar, V., Quillen, D., Gardner, T. W., and Lott, M. E. (2017). Impaired Retinal Vasoreactivity: An Early Marker of Stroke Risk in Diabetes. J. Neuroimaging 27, 78-84. doi: 10.1111/jon.12412.

Conzen, C., Albanna, W., Weiss, M., Kurten, D., Vilser, W., Kotliar, K., et al. (2018). Vasoconstriction and Impairment of Neurovascular Coupling after Subarachnoid Hemorrhage: a Descriptive Analysis of Retinal Changes. Transl. Stroke Res. 9, 284-293. doi: 10.1007/s12975-017-0585-8.

Csipo, T., Lipecz, A., Mukli, P., Péterfi, A., Szarvas, Z., Ungvari, A., et al. (2024). Advancing prediction of age-related vascular cognitive impairment based on peripheral and retinal vascular health in a pilot study: a novel comprehensive assessment developed for a prospective workplace-based cohort (The Semmelweis Study). GeroScience 10.1007/s11357-024-01447-y. Advance online publication. doi: 10.1007/s11357-024-01447-y.

Kotliar, K., Hauser, C., Ortner, M., Muggenthaler, C., Diehl-Schmid, J., Angermann, S., et al. (2017). Altered neurovascular coupling as measured by optical imaging: a biomarker for Alzheimer's disease. Sci. Rep. 7, 12906. doi: 10.1038/s41598-017-13349-5.

Mroczkowska, S., Benavente-Perez, A., Patel, S., Qin, L., Bentham, P., and Gherghel, D. (2014). Retinal vascular dysfunction relates to cognitive impairment in Alzheimer disease. Alzheimer Dis. Assoc. Disord. 28, 366-367. doi: 10.1097/WAD.0b013e3182a2e221.

Querques, G., Borrelli, E., Sacconi, R., De Vitis, L., Leocani, L., Santangelo, R., et al. (2019). Functional and morphological changes of the retinal vessels in Alzheimer's disease and mild cognitive impairment. Sci. Rep. 9, 63. doi: 10.1038/s41598-018-37271-6.

Rensma, S. P., van Sloten, T. T., Houben, A., Kohler, S., van Boxtel, M. P. J., Berendschot, T., et al. (2020). Microvascular Dysfunction Is Associated With Worse Cognitive Performance: The Maastricht Study. Hypertension 75, 237-245. doi: 10.1161/HYPERTENSIONAHA.119.13023.

Szegedi, S., Dal-Bianco, P., Stogmann, E., Traub-Weidinger, T., Rainer, M., Masching, A., et al. (2020). Anatomical and functional changes in the retina in patients with Alzheimer's disease and mild cognitive impairment. Acta Ophthalmol. 98, e914-e921. doi: 10.1111/aos.14419.

van Dinther, M., Voorter, P. H. M., Schram, M. T., Berendschot, T., Houben, A., Webers, C. A. B., et al. (2022). Retinal microvascular function is associated with the cerebral microcirculation as determined by intravoxel incoherent motion MRI. J. Neurol. Sci. 440, 120359. doi: 10.1016/j.jns.2022.120359.
